# Editorial: Emerging Technologies Powering Rare and Neglected Disease Diagnosis and Theraphy Development

**DOI:** 10.3389/fphar.2022.877401

**Published:** 2022-04-05

**Authors:** Zhichao Liu, Qais Hatim, Shraddha Thakkar, Ruth Roberts, Tieliu Shi

**Affiliations:** ^1^ Division of Bioinformatics and Biostatistics, National Center for Toxicological Research, US Food and Drug Administration, Jefferson, AR, United States; ^2^ Office of Translational Sciences, Center for Drug Evaluation and Research, US Food and Drug Administration, Silver Spring, MD, United States; ^3^ ApconiX, BioHub, Alderley Park, United Kingdom; ^4^ Department of Biosciences, University of Birmingham, Birmingham, United Kingdom; ^5^ The Center for Bioinformatics and Computational Biology, The Institute of Biomedical Sciences and School of Life Sciences, Shanghai, China; ^6^ School of Statistics, Key Laboratory of Advanced Theory and Application in Statistics and Data Science-MOE, East China Normal University, Shanghai, China

**Keywords:** emerging technologies, rare diseases, drug repurposing, NGS—next generation sequencing, natural history

Although individual rare disease only affects a very small proportion of populations (i.e., usually less than 1:1500 in the United States or 1:2,000 in Europe), approximately 4% of the total world population experiences rare diseases collectively (Delavan et al., 2018). Obstacles such as incomplete knowledge of natural history hinder the etiology and pathogenesis understanding of rare and neglected diseases. Emerging technologies, including Artificial Intelligence (AI), advanced bioengineering, and next-generation sequencing (NGS), provide unprecedented opportunities to accelerate rare and neglected disease diagnosis and treatment development (Liu et al., 2019). The last day of February every year (i.e., rare disease day) raises awareness of rare diseases and promotes better diagnosis and treatment for eliminating the suffering of rare disease patients. It is an excellent opportunity to highlight this research topic for some recent progress and promising approaches to improve the diagnosis and therapy development of rare diseases. There are 20 papers collected in this research topic, covering a wide spectrum of research in rare diseases, including (1) promising application of NGS technologies in rare disease diagnosis; (2) clinical benefit of NGS-based genetic testing in rare diseases; (3) Genetic variant interpretation; (4) standardization of bioinformatics pipelines in NGS data analysis; (5) rare disease treatment development; and (6) natural history studies for better understating of etiology and pathogenesis of rare diseases.

More than 80% of rare diseases are of genetic origin. NGS-based causal genetic variant detections are indispensable for uncovering the etiology and pathogenesis of rare diseases and potentially facilitating treatment development. Ma et al. conducted a comprehensive genetic analysis based on 28 Chinese families with tyrosinase-positive oculocutaneous albinism (OCA) to identify genetic variants related to *OCA2* gene using NGS and confirmed the findings with sanger sequence. The causal association between genetic variants and clinical outcomes was discussed. Yang et al. employed whole-genome sequencing (WGS) and RNAseq to identify genetic variants that are responsible for peripheral neuroblastic tumors (PNTs) oncogenesis in a trio sample, finding a novel germline compound heterozygous mutation of the BRCA2 gene associated with familial PNTs.

NGS has changed the landscape of genetic detection, providing unprecedented speed and resolution for uncovering individual genetic makeup. Clinical adoption of NGS has been significantly improved to offer diagnostic benefits for rare disease patients. Li et al. showed the potential utility of NGS on cerebrospinal fluid (CSF) for the diagnosis of cerebral alveolar echinococcosis (AE) in a patient with negative biopsy results in conventional clinical diagnosis procedure. Given that the patient had repeat seizures and progressive headaches for only 6 months and repeated target biopsy on the masses in the lung and liver showed fibrous connective tissue without positive findings, physicians successfully utilized the NGS results to diagnose the AE disease. They provided a “fit-for-purpose” treatment regimen (i.e., albendazole therapy). Liu et al. reported a case of a 1-year-old boy with metabolic acidosis and hypokalemia, a small penis, growth retardation, and G-6PD deficiency, whose clinical symptoms were complex and seemingly uncorrelated. The authors employed exome sequencing to identify the genetic mutation based on the trio-sample of the boy’s family and found some *de novo* missense mutation that could be pathologic. Prader–Willi syndrome (PWS) is a complex genetic syndrome caused by the loss of function of genes in 15q11-q13 that are subject to regulation by genomic imprinting and expressed from the paternal allele only. Zhang et al. successfully implemented the preimplantation genetic diagnosis (PGD) to exclude imprinting deficiency in preimplantation embryos before transferring them into the mother’s uterus. The lessons learned from the process further facilitate clinical utility of the procedure.

Complex structural variants (SVs) contribute to a large proportion of human genomic variation and cause rare diseases. Although NGS technologies, especially long-read-based sequencers, have tremendously increased SV detection sensitivity, the inconsistent results yielded by different bioinformatics pipelines still exist. Therefore, more studies on the pros and cons of varying bioinformatics pipelines are urgently needed to standardize the calling practice. Guo et al. conducted a comprehensive comparative study of three popular calling algorithms (i.e., PBSV, Sniffles, and PBHoney) on the GIAB benchmark samples, suggesting that all three pipelines exhibited better performance outside the tandem repeat regions (TRRs) than that of within TRRs. Sun et al. investigated the diagnosis power of the clinical WES technology in Epileptic encephalopathies (EEs) with an in-house pipeline for SNV identification and CNVkit for CNV detection. They found that WES can identify both SNVs/Indels and CNVs in a single flow cell and achieve a high diagnosis rate, suggesting that WES may serve as a first-tier diagnosis tool with cost-effective benefit in EEs.

Genetic variant interpretation and counseling are critical steps to enhance the clinical adoption of NGS testing. Feng et al. presented a case of a girl with non-ketotic hyperglycinaemia (NKH) and associate her genetic variants with clinical manifestations such as spasticity and bilateral cavitating leukoencephalopathy and a deficiency of a respiratory chain enzyme to provide proper genetic counseling. Gong et al. presented the detailed clinical features, such as infantile spasms (IS) and possibly the epilepsy of infancy with migrating focal seizures (EIMFS), and associated *KCNT2* gene mutations in developmental and epileptic encephalopathies (DEEs) to exemplify the genotype-phenotype association analysis in the clinical setting. Alpers’ syndrome is an early inceptive neurodegenerative disorder with a poor prognosis, characterized by developmental regression, intractable epilepsy, and hepatic dysfunction. Li et al. deciphered the electroencephalogram (EEG) characteristics and clinical phenotype of different genotypes of the Alpers’ syndrome and provided clues for early diagnosis. Jiao et al. identified a *de novo* variant (*p.Gly180Trp*) in the *SCAMP5* gene from four unrelated patients with epilepsy and neurodevelopmental delay, elucidating their phenotypic similarity and the difference of onset time, etc. Wang et al. presented the relationship between the patients’ clinical features and the genetic variants in these patients identified by WES with a comprehensive literature survey, clarifying the mutational characteristics of causative genes in Hereditary spherocytosis (HS).

Natural history studies play a critical role in understanding the etiology, range of manifestations, and progression of rare diseases. Liu et al. carried out a meta-analysis of 36 encephalopathy patients. They identified a novel *de novo* genetic variant (i.e., (*c.116G>A*, *g.22229G>A*, *p.S39N*) in the GTPase domain of *DNM1L*) and elucidated its association with mitochondrial-related clinical phenotypes. Xu et al. investigated the pathogenicity of the novel mtDNA variant m.9396G > A/MT-CO3 (*p.E64K*) in a patient with mitochondrial encephalomyopathy, lactic acidosis, and stroke-like episodes (MELAS) with biochemical assays. They found a muscle biopsy which confirmed the remarkable CIV deficiency caused by the novel mutation, characterized by ragged red fibers and generalized COX non-reactive muscle fibers. Hong et al. used a rat model to investigate the role of *GABRD* gene methylation in the nucleus accumbens in heroin-seeking behavior and suggested a potential treatment option to treat heroin addiction and relapse. Yang et al. revealed the association between a sex chromosome mosaicism present in the oral epithelial cells or gonad tissues of patients with sex development disorder (DSD) and with XYY in blood using Fluorescence *in situ* hybridization (FISH), enhanced the understanding of the genetic basis of DSD in males.

There are over 7,000 rare diseases. However, only less than 700 treatment options are available. There are tremendous unmet needs for rare disease treatment development. Sanchez et al. reviewed the progress of Alagille Syndrome (ALGS) treatment development, emphasized the emerging technologies such as NGS and advanced bioengineering in the development of targeted therapies for ALGS. Zhou et al. proposed actionable clinical decisions for CAD deficiency with specific manifestation such as co-occurrence of drug-resistant epilepsy, mainly focal or with generalization. They emphasized the need to act immediately upon discovering such variants in treatable disease genes. Wang et al. compared the eight patients with 3-Hydroxyisobutyryl-CoA hydrolase (HIBCH) deficiency to summarize the common clinical features caused by HIBCH genetic mutation. The outlined common clinical features further guided the treatment regimen strategy development, resulted in the improved clinical outcome of HIBCH patients. Liu et al. utilized the gene fusion profiles of neuroblastoma (NB) patients to stratify the patients into newly defined subgroups with increased survival curves. Furthermore, the authors also carried out the precision medicine-based drug repurposing to the refined high-risk patient subgroups to optimize the treatment regimens.
